# Growth inhibition and apoptosis induction of *Scutellaria luteo-coerulea Bornm.* & Sint. on leukemia cancer cell lines K562 and HL-60

**Published:** 2015

**Authors:** Mahsa Motaez, Seyed Ahmad Emami, Zahra Tayarani-Najaran

**Affiliations:** 1 *Department of Pharmacodynamics and Toxicology, School of Pharmacy, Mashhad, University of Medical Sciences, Mashhad, Iran*; 2 *Department of Pharmacognosy, School of Pharmacy, Mashhad, University of Medical Sciences, Mashhad, Iran*

**Keywords:** *Scutellaria*, *luteo-coerulea*, *Cytotoxicity*, *Apoptosis*, *Lamiaceae*

## Abstract

**Objective:**

*Scutellaria* (Lamiaceae) has been implicated for medicinal purposes both in modern and traditional medicine. Some species of the genus *Scutellaria* has extensively been studied for anticancer activity. *Scutellaria luteo-coerulea *(*S. luteo-coerulea*) is one of the Iranian species of the genus *Scutellaria*.

**Materials and Methods:**

In the present study, cytotoxic and apoptogenic properties of CH_2_Cl_2_, EtOAc, *n*-BuOH, and H_2_O fractions of *S. luteo-coerulea *were investigated on K562. Moreover, HL-60. DNA fragmentation in apoptotic cells were determined by propidium iodide (PI) staining (sub-G1 peak).

**Results:**

*Scutellaria* *luteo-coerulea* inhibited the growth of malignant cells in a dose-dependent manner. Among solvent fractions of *S.* *luteo-coerulea*, the CH_2_Cl_2_ fraction was found to be the most cytotoxic one among others. Sub-G1 peak in flow cytometry histogram of treated cells suggested the induction of apoptosis in *S. luteo-coerulea*.

**Conclusion:**

*Scutellaria luteo-coerulea *could be a novel candidate for further analytical elucidation in respect to fine major components responsible for the cytotoxic effect of the plant also clinical evaluations.

## Introduction

The genus *Scutellaria *(Lamiaceae) with about 360 different species, is one of the popular herbs which has been extensively investigated in biological researches. Some plants of the genus *Scutellaria *have been used in traditional and folk medicine. Since 1889, phenolic (flavonoids, phenylethanoid glycosides) and terpene compounds (iridoid glycosides, diterpenes, and triterpenoids) have been identified as two main groups of constituents of *Scutellaria*. Baicalin, baicalein, wogonin, and neobaicalein are among the main compounds of flavonoids possess anti-cancer, anti-microbial, anti-inflammatory, and anticonvulsant effects (Shang et al., 2010 ▶). Baicalein and baicalin flavonoids were shown to have protective properties against reactive oxygen species (ROS)-induced tissue damage and antimicrobial activity. The inhibition of HIV-1 reverse transcriptase has also been reported for baicalein (Mamadalieva et al., 2011 ▶). Wogonin, baicalein and baicalin as the major constituents of *S. baicalensis* have been shown cytotoxic activity to various human tumor cell lines with minor toxicity to normal cells (Li-Weber, 2008 ▶). Neobaicalein an active ingredient of the *S. litwinowii* exerts cytotoxic and pro-apoptotic effects in human cervical cancer, HeLa cell line (Tayarani-Najarani et al., 2012 ▶).

*Scutellaria colebrookiana* and *S. violacea* showed cytotoxic and antioxidant potential which may attributed to the presence of baicalein (Salini et al., 2013 ▶). *Scutellaria baicalensis* and* S. rivularis* have been revealed inhibitory effect on HL-60 cells which is related to flavonoids in the plant (Sonoda et al., 2004 ▶).

Baikal skullcap (*S. baicalensis*) and *S. barbata* possess anti-proliferative and apoptosis-inducing activities (Yu et al., 2007 ▶; Kim et al., 2007 ▶). *Scutellaria* radix has an anti-proliferative effect on myeloma cells and baicalein may be responsible for the inhibitory activity of *Scutellaria* radix by blocking IkB-degradation (Ma et al., 2005 ▶). Inhibition of growth of different tumor cell lines by flavones,i.e., wogonin, baicalein, and baicalin as well as stimulation of apoptosis of cancer cells were reported as the most important anticancer reaction of *Scutellaria* flavones (Lamer-Zarawska et al., 2010 ▶). *Scutellaria* *baicalensis* has also had strong inhibitory effect on cell growth in prostate and breast cancer cells (PC-3, LNCaP, and MCF-7) which may partly be due to its ability to inhibit COX-2 activity (Ye et al., 2002 ▶). 

In the course of cytotoxic activity stated for several *Scutellaria* species, the present paper describes the anti-proliferative and apoptogenic activity of *S. luteo-coerulea*, one of the Iranian species of *Scutellaria*.

There is not any reported literature on* S. luteo-coerulea*. In order to give insight to potential cytotoxic activity of the *S. luteo-coerulea*, CH_2_Cl_2, _and EtOAc, *n*-BuOH and H_2_O fractions of the plant have been evaluated on K562 and HL-60 cells. The role of apoptosis in this cytotoxicity was also assessed.

## Materials and Methods

**Reagents and chemicals**

RPMI-1640 medium and fetal bovine serum were purchased from Gibco (London, UK); 3- (4,5-dimethylthiazol-2-yl) -5- (3-carboxymethoxyphenyl) -2- (4-sulphophenyl)-2H-tetrazolium(MTS), from Promega (Madison, WI, USA); Propidium Iodide (PI) from sigma (St. Louis, MO, USA).

**Plant materials**

**Figure 1:**
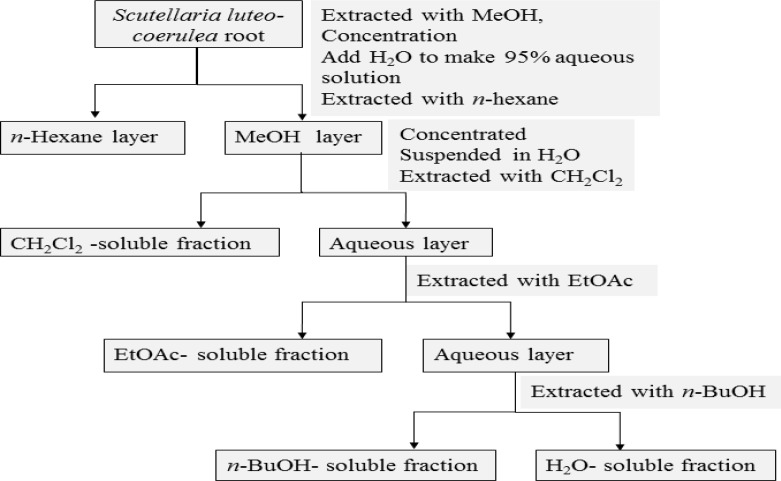
Partitioning scheme using immiscible solvents

Roots of *S. luteo-coerulea* were collected from Tandooreh Natural Park, Daregaz (2100 m height), Razavi Khorasan province, northeast of Iran. The plant was identified by Dr. I. Mehregan (2002). Voucher specimen (No.11318) was deposited in herbarium of School of Pharmacy, Mashhad University of Medical Sciences, Mashhad, Iran. The extraction method was done according to previously reported protocol (Tayarani-Najaran et al., 2013 ▶) (Figure 1.). 

Cytotoxic and apoptosis assays were performed using the isolated fractions in dimethylsulfoxide (DMSO).

**Cell cultures**

The human leukemia cancer cells (K562 and HL-60) were obtained from Pasteure institute (Tehran, Iran) and maintained in RPMI-1640 medium supplemented with 10% fetal bovine serum, 100 U/mL penicillin, and 100 µg/mL streptomycin at 37 ºC in a humidified atmosphere of 95% air and 5% CO_2_. The stock solution of each compound was prepared at 50 mg/mL in dimethylsulfoxide and kept at -20 °C.

For MTS and apoptosis assay, 10^4^ cells/well and 10^5^ cells/well were seeded to 96-well and 24-well plates, respectively. Each assay was compared with a untreated control received the equal volume of DMSO.

**Cell viability**

The reduction of 3- (4,5-dimethylthiazol-2-yl) -5- (3-carboxymethoxyphenyl) -2- (4-sulphophenyl) -2H-tetrazolium inner salt (MTS) by mitochondrial dehydrogenase in live cells produce the colored, water-soluble formazan that absorbs at 490 nm (Malich et al., 1997 ▶). For MTS assay, K562 and HL-60 cells were treated with various concentrations of each fraction of *S. luteo-coerulea*. After 48 h incubation, MTS (Promega, Madison, WI, USA) was added to each well according to the manufacturer’s instructions. After 3 h in culture, cell viability was determined by measuring the absorbance using an ELISA microplate reader (Awareness, Palm City, FL, USA). The cytotoxicity of CH_2_Cl_2_ and EtAOc fractions of *S. luteo-coerulea* was expressed as IC_50_, which was calculated using Graph Pad software (Graph Pad prism 5 software) and presented as mean±SEM of three independent experiments with three replicates for each concentration fraction of *S. luteo-coerulea *fractions.

**PI staining**

PI stained cells were evaluated by flow cytometry to detect the so-called sub-G1 peak in apoptotic cells (Nicoletti et al., 1991 ▶). Briefly, 10^5 ^K562 and HL-60 cells were seeded in each well of a 24-well plate and treated with CH_2_Cl_2_ fraction of *S. luteo-coerulea* different concentrations (0-125 µg/mL) for 48 h. Floating and adherent cells were then harvested and incubated with 50 μg/mL PI in 0.1% sodium citrate plus 0.1% Triton X-100. Small fragments of DNA in apoptotic cells were eluted following incubation in a hypotonic phosphate-citrate buffer and stained with PI before flow cytometric analysis using a FACScan flow cytometer (Becton Dickinson) was done. 10^4^ events were acquired with FACS. 

**Statistical analysis**

One way analysis of variance (ANOVA) and Bonferroni’s post hoc were used for data analysis. All results were expressed as mean±SEM and *p* values below 0.05 were considered statistically significant. 

## Results

**Inhibition of cell viability**

Inhibition of cell viability caused by fractions extract of *S. luteo-coerulea* was examined using MTS assay. 

**Figure 2:**
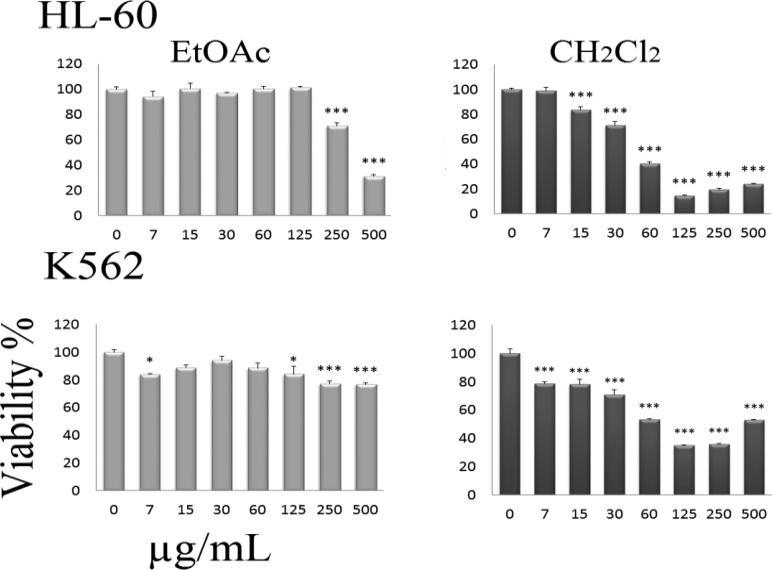
Dose-dependent growth inhibition of K562 and HL-60 cells by solvent fractions of *S. luteo-coerulea *(0-500 µg/mL) after 48 h. Viability was quantitated by MTS assay. The dose inducing IC_50_ against K562 and HL-60 by CH_2_Cl_2_, EtOAc, solvent fractions of *S. luteo-coerulea* were calculated 94.39, 1093, 53.96, and 528.8 respectively. Paclitaxel (700 nM) was used as a positive control. Results are expressed as mean±SEM (n = 3). ∗p<0.05, ∗∗p<0.01, and ∗∗∗p<0.001 compared to control.

In order to compare the cytotoxicity of fractions extract of *S. luteo-coerulea* the K562 and HL-60 cells were incubated with different concentrations for 48 h. The results showed these compounds decreased cell viability of cells in a concentration-dependent manner (Figure 2). The doses inducing 50% cell growth inhibition (IC_50_) against K562 and HL-60 cells for CH_2_Cl_2_ and EtOAc fractions extract of *S. luteo-coerulea* are presented in Table 1. We used 700 nM of Paclitaxel as a positive control.

**Table 1:** IC_50_ values (µg/mL) for different solvent fractions of *S. luteo-coerulea* in K562 and HL-60 cell lines.

**Cell Line**	**Fraction**	**IC**_50_	**IC**_50 _ **range**
**K562**	CH_2_Cl_2_	94.39	38.14 to 233.6
	EtOAc	1093	499.4 to 2393
**HL-60**	CH_2_Cl_2_	53.96	31.04 to 93.80
	EtOAc	528.8	232.7 to 1202

**Apoptosis induction by CH**_2_ **Cl**_2_ **fraction in K562 and HL-60 cells**

**Figure 3:**
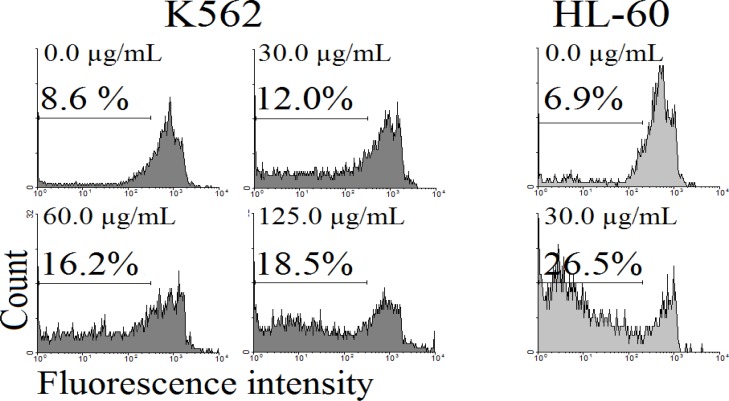
Flow cytometry histograms of apoptosis assays by PI method in K562 and HL-60 cells. Cells were treated with different concentration of CH_2_Cl_2_ solvent fractions of *S. luteo-coerulea *(0, 15, 30, 60, and 125 µg/mL) for 48 h. Sub-G1 peak as an indicative of apoptotic cells, was induced in CH_2_Cl_2_ solvent fractions of *S. luteo-coerulea *treated but not in control cells. CH_2_Cl_2_ fraction-treated cells exhibited a sub-G1 peak in K562 and HL-60 cells in a concentration-dependent manner that indicates the involvement of an apoptotic process in CH_2_Cl_2_ fraction-induced cell death

Cells treated with CH_2_Cl_2 _solvent fraction of *S. luteo-coerulea* were stained with PI and the percentage of apoptotic cells following treatment was measured by flow cytometry aiming to detect the sub-G1 peak resulting from DNA fragmentation. The K562 and HL-60 cells treated with 15, 30, 60, 125, and 250 µg/mL CH_2_Cl_2_ solvent fraction of *S. luteo-coerulea *for 48 h induced a sub-G1 peak in flow cytometry histogram compared to untreated control cells (Figure 3).

## Discussion

The anti-proliferative and cytotoxic effect of many *Scutellaria* species on several cancer cell lines have been reported previously (Salini et al., 2013 ▶; Tayarani-Najaran et al., 2010 ▶; Tayarani-Najarani et al., 2012 ▶). In the present study, the cytotoxic properties of fractions of *S. luteo-coerulea* were investigated on K562 and HL-60. Fractions were tested for cytotoxic activity for range of 0-500 μg/mL after 48 h of treatment. 

Wang et al., reported potent cytotoxic activities of eight crude extracts of five *Scutellaria* species against brine shrimps and four human cancer cell lines (HCA, HepG2, MCF-7, HPC) (Wang et al., 2010 ▶). In another study, *in vitro* anti-proliferative screening investigation of crude methanol extracts of *S. dominica* L. leaves, *S. lanigera *Desf. aerial parts, *S. menthaefolia* Ten. roots, *S. palaestina *Benth. aerial parts, *S. sclarea* L. roots, and *S. spinosa* L. aerial parts have shown cytotoxic effects with IC_50_ values ranged from 90 to 400 μg/mL (Sonoda et al., 2004 ▶).

Different researches have been performed on active component of the species including baicalin, baicalein, and neobaicalein. Baicalein inhibited the proliferation of estrogen receptor positive human breast cancer MCF-7 cells in vitro (Wang et al., 2010 ▶). Baicalin and neobaicalein were found to induce apoptosis in human promyelocytic leukemia, HL-60, and HeLa cells, respectively through multiple pathways and may be an interesting strategy in treatment of leukemia. Pro-apoptotic mechanism of baicalin in leukemia Jurkat cells has been verified with characteristic morphologic change of apoptosis (Lamer-Zarawska et al., 2010 ▶). Baicalin also presented antiviral activity against viruses which have been identified as carcinogens. The anti-inflammatory effect of the *Scutellaria *flavones via down-regulation of several inflammation-associated genes in malignant tumors is known to exert both pro- and antitumor effects (Lamer-Zarawska et al., 2010 ▶).

In our study, fractions obtained from *S. luteo-coerulea* had cytotoxic and apoptotic activities on K562 and HL-60 cells. In order to gain insight into the nature of the active principles responsible for the cytotoxic activity, the methanol extract was fractionated using solvents of increasing polarity. The IC_50_ values of the fractions are compared in Table 1. Active constituent(s) of intermediate polarity is thus likely to be responsible for the observed cytotoxicity and future bio-assay guided fractionation needs to focus on the CH_2_Cl_2_ fraction. These results suggest that CH_2_Cl_2_ fraction obtained from *S. luteo-coerulea *could be used as a potential apoptosis inducing agent, and that the CH_2_Cl_2_ fraction obtained from *S. luteo-coerulea* consists of a key component for cytotoxic activity. Regarding the sequential extraction with solvents of ascending polarity, the fraction of *S. luteo-coerulea* with CH_2_Cl_2_ generated fractions that were cytotoxic for tumor cells. This can be explained by the low polarity of the solvents used in these fractions, which extract low-polar compounds that are either successfully absorbed through the cell or have cytotoxic activity. Therefore, it is presumed that most of the cytotoxic compounds or compounds with the highest potency were concentrated in the CH_2_Cl_2_ fraction.

In conclusion, this study determined an anti-cancer effect of CH_2_Cl_2_ fraction obtained from *S. luteo-coerulea *mediated by the induction of apoptosis, which is associated with DNA fragmentation in K562 and HL-60 cells. 

Due to potential value of apoptosis in cancer therapeutic strategies, these results confirm the possible worth of CH_2_Cl_2 _fraction obtained from *S. luteo-coerulea* as cytotoxic agents. CH_2_Cl_2_ fraction can extract a compound that has considerable cytotoxic activity. However, further investigations on the active principals of CH_2_Cl_2_ fraction obtained from *S. luteo-coerulea *are necessary to be elaborated.
